# Preliminary effect and feasibility of physiotherapy with strength training and protein-rich nutritional supplement in combination with anabolic steroids in cross-continuum rehabilitation of patients with hip fracture: protocol for a blinded randomized controlled pilot trial (HIP-SAP1 trial)

**DOI:** 10.1186/s13063-019-3845-y

**Published:** 2019-12-23

**Authors:** Signe Hulsbæk, Ilija Ban, Tobias Kvanner Aasvang, Jens-Erik Beck Jensen, Henrik Kehlet, Nicolai Bang Foss, Thomas Bandholm, Morten Tange Kristensen

**Affiliations:** 1 Physical Medicine and Rehabilitation Research - Copenhagen (PMR-C), Department of Physiotherapy, Copenhagen University Hospital, Amager-Hvidovre, Kettegård Alle 30, 2650 Hvidovre, Denmark; 2 Department of Orthopedic Surgery, Copenhagen University Hospital, Amager-Hvidovre, Kettegård Alle 30, 2650 Hvidovre, Denmark; 3 Department of Endocrinology, Copenhagen University Hospital, Amager-Hvidovre, Kettegård Alle 30, 2650 Hvidovre, Denmark; 4 Department of Clinical Medicine, University of Copenhagen, Copenhagen, Denmark; 5 Section for Surgical Pathophysiology 721, Copenhagen University Hospital, Rigshospitalet Ole Maaløes vej 26, 2100 Copenhagen Ø, Denmark; 6 Department of Anesthesiology, Copenhagen University Hospital, Amager-Hvidovre and Institute of Clinical Medicine, University of Copenhagen, Kettegård Alle 30, 2650 Hvidovre, Denmark; 7 Clinical Research Centre, Copenhagen University Hospital, Amager-Hvidovre, Kettegård Alle 30, 2650 Hvidovre, Denmark

**Keywords:** Hip fracture, Rehabilitation, Physiotherapy, Strength training, Nutritional supplement, Protein, Anabolic steroid

## Abstract

**Background:**

A 2014 Cochrane review evaluating the effect of anabolic steroids after hip fracture concluded that the quality of the studies was insufficient to draw conclusions on the effects and recommended further high-quality trials in the field. Therefore, the aim of this pilot trial is to determine the preliminary effect and feasibility of a 12-week multimodal intervention consisting of physiotherapy (with strength training), protein-rich nutritional supplement and anabolic steroid on knee-extension muscle strength and function 14 weeks after hip fracture surgery.

**Methods:**

We plan to conduct a randomized, placebo-controlled pilot trial with 48 patients operated for acute hip fracture. The patients are randomized (1:1) to either (1) physiotherapy with protein-rich nutritional supplement plus anabolic steroid or (2) physiotherapy with protein-rich nutritional supplement plus placebo. Outcome assessments will be carried out blinded at baseline (3–10 days after surgery) and at 14 weeks after entering the trial. Primary outcome is the change from baseline to follow-up in maximal isometric knee-extension muscle strength in the fractured limb. Secondary outcomes are physical performance test, patient-reported outcomes, and measures of body composition.

**Discussion:**

If the trial is found feasible and the results show an indication of anabolic steroid being a relevant addition to further enhance the recovery of muscle strength and function in an enhanced recovery after surgery program, this trial will constitute the basis of a larger confirmatory trial.

**Trial registration:**

ClinicalTrials.gov, NCT03545347. Preregistered on 4 June 2018.

## Background

Sustaining a hip fracture is a common event with major consequences for the individual and society. Northern Europe has the highest incidence rates, led by Denmark, with age-standardized annual rates of 574 per 100,000 in women and 290 per 100,000 in men [1]. Furthermore, incidence rates are expected to increase worldwide due to the aging populations [2].

Patients sustaining a hip fracture experience an immediate loss of knee-extension muscle strength in the fractured limb [3–5]. Decreased lower limb muscle strength is associated with impaired function and disability [4, 6, 7], and it is an independent predictor of falls within 6 months of the hip fracture [8]. As such, a hip fracture often leads to loss of independence, change of residence, further fractures, and high mortality rates [9–13]. Thus, hip fractures pose a substantial economic burden to the health care system and society in general [2, 14, 15].

The evidence regarding rehabilitation following hip fracture shows positive effects on mobility after structured exercise interventions including progressive strength training [16–19]. However, these interventions are mainly started months after the hip fracture has occurred as prolonged programs following ceased standard rehabilitation [16–19]. This is costly and does not reflect the usual standard rehabilitation program offered to patients with hip fracture [20]. On the other hand, although a positive effect of structured exercise has been shown, it seems that these interventions alone are insufficient to overcome the major long-term negative impact of a hip fracture on physical function [10].

A recent (2014) Cochrane systematic review has evaluated the effect of anabolic steroids, either separately or in combination with nutritional supplements, in rehabilitation following hip fracture surgery in terms of functional outcome and adverse events (AEs) [21]. Although positive tendencies were identified in relation to activities of daily living and hip-related function [22, 23], quality of life [22], gait speed [23], and reduction in loss of muscle mass [22, 23], the quality of the studies was insufficient to draw definitive conclusions on the effect [21]. It was emphasized that further high-quality trials are warranted [21], and this is supported by several other narrative reviews in the field [6, 13, 24].

Another common and ongoing challenge to optimal recovery after hip fracture and hospitalization is low protein intake in elderly patients [25]. A recent Cochrane systematic review of the effect of nutritional supplementation for older patients recovering from hip fracture concluded that there might be some effect in relation to reducing complications within the first 12 months, but the evidence is weak [26].

On the basis of our previous early exercise studies [3, 27, 28] and review recommendations [6, 13, 16, 21, 25], it seems rational and strongly needed to apply an early multimodal intervention consisting of muscle-building medicine as well as nutritional and physical exercise treatment in order to enhance short- and long-term outcomes after the disabling event of a hip fracture.

### Purpose

The aim of this pilot trial is to investigate the preliminary effect and feasibility of a 12-week multimodal intervention consisting of physiotherapy (functional, balance, and strength training), protein-rich nutritional supplementation, and anabolic steroid (INT) compared with physiotherapy (functional, balance, and strength training), protein-rich nutritional supplement, and placebo (CON) in rehabilitation following hip fracture surgery on fractured limb knee-extension muscle strength at 14-week follow-up.

We hypothesize the following:

1. An intervention consisting of physiotherapy, nutritional supplementation, and anabolic steroid is a feasible and preliminary safe treatment in elderly patients with hip fracture when initiated in the acute orthopedic ward and continued for 12 weeks.
2. This multimodal intervention (physiotherapy, nutritional supplementation, and anabolic steroid) is more efficacious in improving muscle strength and physical function 14 weeks after hip fracture surgery than physiotherapy, nutritional supplementation, and placebo.

## Methods

### Trial design

The HIP-SAP1 trial (Hip fracture, Strength training, Anabolic steroid and Protein) is a randomized, blinded, single-center, placebo-controlled, two-arm, parallel-group, superiority pilot trial. We intend to include 48 patients with hip fracture who will be randomized (1:1) to one of two arms.

This clinical trial protocol is based on the PREPARE trial guide [29], the SPIRIT (Standard Protocol Items: Recommendations for Interventional Trials) checklist (Additional file 1), and the CONSORT (Consolidated Standards of Reporting Trials) checklist (extension to randomized pilot and feasibility trials). The Template for Intervention Description and Replication (TIDieR) checklist [30] is used for description of the intervention. The trial was registered at ClinicalTrials.gov (identifier NCT03545347) before the first participant was included. The trial will be conducted at Copenhagen University Hospital Hvidovre in cooperation with all municipalities in the catchment area of the hospital.

### Recruitment

Patients admitted to the Hip Fracture Unit at the Orthopedic Department of Copenhagen University Hospital Hvidovre will be screened for eligibility (see inclusion and exclusion criteria in Table 1). The sampling method is consecutive, though screening and inclusion will be discontinued during trial staff’s absence. A screening log will be kept. Annually, approximately 475 patients above 60 years of age are operated at the Hip Fracture Unit. We assume that 20% would be excluded due to nursing home residency, another 20% would be excluded due to cognitive impairments, and the remaining criteria would account for approximately 25% exclusions. That would leave us with 14 eligible patients per month. We aimed at completion of recruitment within 1 year, but because recruitment has been lower than expected, the recruitment period has been extended to September 2020.

**Table 1:** Inclusion/exclusion criteria

*Inclusion criteria*	
• Patients who have undergone surgery for a hip fracture at Amager-Hvidovre University Hospital and admitted to the Hip Fracture Unit at the hospital	
• Age ≥ 60 years	
• Ability to speak and understand Danish and having a Danish Social Security number	
• Able to give written informed consent	
• Residing at home and with an independent prefracture indoor walking ability (New Mobility Score ≥ 2)	
*Exclusion criteria*	
• Postoperative weight-bearing restrictions	
• Multiple fractures	
• Active cancer or suspected pathological fracture	
• Patient unable/unwilling to cooperate for testing and rehabilitation	
• Planned/elective hospitalization within the trial period	
• Cognitive dysfunction determined by chart review, reported by nursing staff, or observed by trained research staff (disoriented, dementia, active delirium)	
• Uncontrolled blood pressure (systolic > 150 mmHg or diastolic > 100 mmHg)	
• Heart disease in the form of peri-, myo-, or endocarditis	
• History of stroke with motor disability	
• Heart failure (New York Heart Association class III and IV)	
• Evidence of kidney failure or renal impairment (estimated glomerular filtration rate < 30 ml/min/1.73 m^2^ or serum creatinine > 200 μmol/L)	
• Abnormal liver function tests (alanine aminotransferase, γ-glutamyltransferase, bilirubin, or alkaline phosphatase > 2 times the upper limit of normal) or history of hepatic tumor	
• Elevated hematocrit ≥ 50%	
• History of breast or prostate cancer	
• Abnormally elevated serum prostate-specific antigen (PSA) assessed at the 3-week control* corresponding to PSA < 4.0 μg/L (60–70 years), PSA < 5.0 μg/L (> 70 years)	
• Allergic to any ingredient in the Deca-Durabolin solution (nandrolone, benzyl alcohol, arachis oil [peanut oil], and allergy to peanuts or soya) or milk protein allergy (related to the nutritional drink)	

*PSA during admission could be increased due to catheterization; therefore, PSA will be assessed at 3 weeks, and patients will be excluded at this time point if elevated values are identified

Information regarding inclusion and exclusion criteria will be obtained through medical records and by asking the patient or relatives. Assessment of eligibility is a two-stepped process because hematocrit and liver functions tests are not standard tests following hip fracture surgery, and therefore blood tests cannot be taken until after informed consent has been obtained.

The initial screening for eligibility will be conducted by the project coordinator, and final assessment of patients eligible for inclusion in the trial will be conducted by the principal investigator or two other medical doctors allocated to the hip fracture unit and trained in the protocol. These individuals will all be blinded to the allocation sequence.

Eligible patients will be addressed at the ward by the project coordinator 1–4 days following surgery. Patients will receive full oral and written information from the project coordinator about the purpose of the trial, process, and potential benefits and risks. The information is delivered by the project coordinator in simple language without technical or value-laden terms, and it is given in a considerate manner, tailored to the individual subject. The patients will be offered 24 h to consider participation, and they will be informed of the possibility of having a relative or other person accompanying them for further information. It is ensured that all questions the patient might have are answered. Patients who agree to participate must sign an informed consent form, which the project coordinator also signs for the given information.

Patients will be informed that participation is voluntary and that they can withdraw their consent at any time and leave the trial. It is emphasized that nonparticipation will not affect further treatment at the department.

### Intervention

**Fig. 1:**
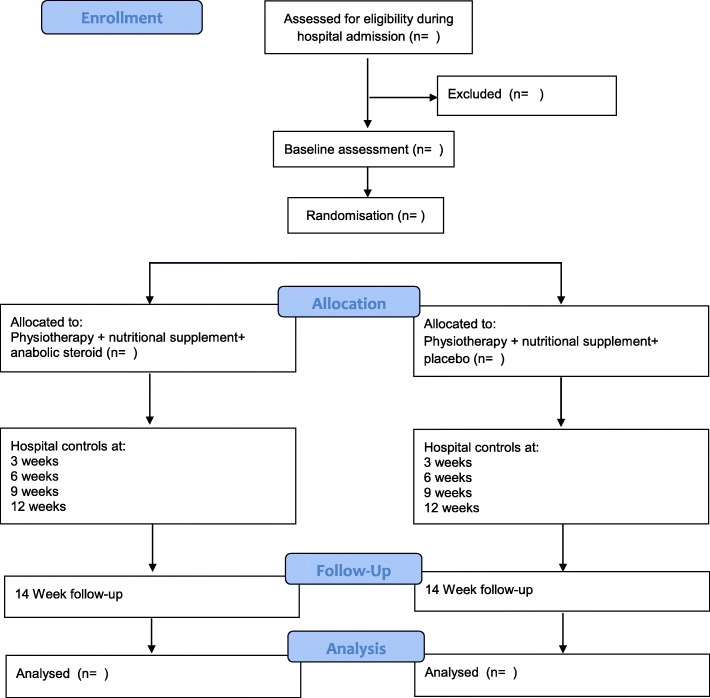
Flowchart of enrollment, randomization, and trial-related activities

After inclusion in the trial, baseline assessments will be performed, and hereafter patients will be randomized (1:1) to one of two arms receiving either (1) physiotherapy with nutritional supplementation and anabolic steroid (INT) or (2) physiotherapy with nutritional supplementation and placebo (CON). See Fig. 1 for a flowchart of the trial.

#### Trial medication

Nandrolone is a synthetic anabolic-androgenic steroid; it is protein-building, promotes mineralization of bones, and stimulates the formation of red blood cells. Nandrolone is structurally related to naturally occurring testosterone, but it shows enhanced anabolic effect and a reduced androgenic effect. Nandrolone is used medically in the form of esters (nandrolone decanoate) and is intended for use in osteoporosis in postmenopausal women and for some types of anemia. Deca-Durabolin is an intramuscularly administered depot preparation of nandrolone decanoate.

- *Active arm (INT)*: Patients will receive intramuscular injections of nandrolone decanoate (Deca-Durabolin 50 mg/ml; Aspen, Durban, South Africa) every 3 weeks. The first injection will be administered at baseline and the last injection at week 12. The solution is injected into the gluteal muscle or rectus femoris. The dosage varies, dependent on gender and testosterone level (men). Women will receive 50 mg, men with total testosterone ≥ 11 nmol/L will receive 100 mg, and men with total testosterone < 11 nmol/L will receive a dose of 200 mg. The cut of 11 nmol/L for total testosterone is determined on the basis of the age-related reference interval (men 50–70 years, 8.4–25.4 nmol/L) and lies below the mean value of 14.6 nmol/L (24).
- *Placebo arm (CON)*: Patients will receive a placebo injection of 1 ml of sodium chloride 9 mg/ml (produced by Fresenius Kabi, Bad Homburg, Germany), following the same intervals as for the active agent. The fluid is injected intramuscularly, and the product has no therapeutic effect.

#### Nutritional supplementation

Patients in both arms will receive two daily nutritional drinks while under hospital admission, which is already a standard procedure at the unit. At discharge, patients will receive nutritional drinks covering the following 3 weeks. At every control visit at the hospital, additional drinks will be provided covering the next 3 weeks.

The protein-rich nutritional supplement is planned to account for at least 35% of the patient’s daily protein requirement. The recommendations for geriatric patients with acute disease is 1.2–1.5 g/kg body weight/day [31]. The standard used at the hip fracture unit is 1.35 g/kg body weight/day; this value will be used to calculate the protein supplementation throughout the study. The protein-rich nutritional supplement is a liquid containing 18 g of milk-based protein per bottle (RESOURCE 2.0 + fiber; Nestlé Health Science, Sydney, Australia). On the basis of the standard used in this study, dependent on their body weight, most patients will receive two bottles per day for 12 weeks.

#### Physiotherapy

Patients will receive physiotherapy as part of the department’s standard procedure, starting on the day after surgery and continued daily until day 3 postoperatively, and thereafter continued on weekdays. The standard physiotherapy treatment includes functional exercises, such as transfers and walking, and exercise therapy primarily aimed at lower extremities. An exercise guideline with 12 specific exercises focusing on joint movement, lower limb muscle activation, and edema prophylaxis will be handed out and progressed individually [3]. After baseline testing and randomization, knee-extension strength training using weight cuffs will be added. The intervention is adjusted to meet the abilities of the individual patient, considering their medical and prefracture status. On days of baseline testing, the testing replaces the normal physiotherapy intervention.

After discharge, patients will receive physiotherapy in the municipality, which is already a standard procedure in Denmark following a hip fracture. The patients will receive physiotherapy 1 h twice weekly up to and including the 12th week after inclusion in the study. The physiotherapy intervention in the municipality will typically be a group intervention, and it will be based on the patient’s individual level. The training will consist of a warmup (aerobic exercises such as cycling), functional training (e.g., walking exercises, climbing stairs, sit-to-stand exercise), balance training (with different degrees of support and different types of underlay), and lower limb exercises (e.g., using elastic bands and progressive strength training). In regard to strength training, two exercises will be obligatory (knee extension performed as unilateral and bilateral leg press), which will be performed according to a standardized protocol (Additional file 2). Patients will perform three sets for each exercise. During the first 2 weeks, the exercises will be performed with approximately 15 repetitions (reps) and an intensity of 15 repetition maximum (RM), and thereafter 2 weeks of 12 reps with 12 RM, and for the remaining 8 weeks 10 reps with 10 RM [28]. The physiotherapist will log the load, repetitions, and pain for each set during the session and progress the load on a set-to-set basis. The patient is instructed to take as many repetitions as possible in each set; if the number of repetitions varies by more than 3 in relation to the number planned, then the load will be adjusted. Both concentric and eccentric phases are performed slowly and in a controlled manner (see Additional file 2 for exercise log).

The engaged physiotherapists in the municipalities are experienced and have been involved in the process of designing/describing the physiotherapy intervention. Prior to initiating the trial, the primary author visited the sites to ensure consistency across the nine rehabilitation centers.

#### General trial treatment procedures

Patients included in the trial will be treated according to the department’s standard procedures for surgery, anesthesia, and perioperative care. Type of operation is determined by a well-defined algorithm based on the type of fracture [32]. Standard perioperative care includes D vitamin and calcium supplementation dependent on the patient’s individual level. Further, a standardized liberal transfusion protocol is used with transfusion if hemoglobin (Hb) is < 9.7 g/dl.

After enrollment, patients will be assessed in regard to the study’s primary and secondary outcomes. Thereafter patients are randomized, and the first injection of the trial solution is administered by the dedicated nurse. In case of the primary nurse being absent, a second nurse trained in the protocol will substitute for her.

After discharge, the patient will receive weekly telephone calls from the project coordinator in order to ensure and monitor compliance. The patient will be asked about the amount of consumed nutritional supplement and attendance at physiotherapy sessions. Further, the patient will be asked about their well-being in order to detect potential side effects of the intervention. An interview guide will be used to assure systematic collection of information.

Every 3 weeks, the patient will attend a control visit at the hospital. The dedicated nurse will carry out blood tests and inject the treatment solution according to randomization group. Compliance with the trial as well as outcome/safety parameters will be monitored. Further, the nutritional supplement covering the following 3 weeks will be handed out at the visit.

The intervention period for the nutritional supplementation and exercise intervention is 12 weeks. The patient will receive the last injection at week 12. Further, at the 12-week appointment, an activity monitor will be applied to the patient’s thigh (activPAL; PAL Technologies, Glasgow, UK), which will monitor activity the following week.

Follow-up will occur during week 14, when patients will be assessed according to the primary and secondary outcomes. To ensure that all potential side effects are detected, one last telephone call is conducted during week 16, and thereafter the patient will have no further obligations in relation to the trial. See Fig. 1 for the flow of enrollment and trial-related events.

### Criteria for discontinuation

Safety parameters are listed under the “Secondary outcomes” heading and will be observed throughout the study. If values of hematocrit, liver tests, and prostate-specific antigen (PSA) exceed the safety thresholds, the treatment with Deca-Durabolin will be discontinued. Further, if women experience androgenic side effects, treatment will be discontinued. Regarding the remaining safety parameters, where no safety threshold is specified, values outside the reference interval will be evaluated by the medical doctors trained in the protocol and relevant action will be taken if necessary.

In accordance with the Declaration of Helsinki, patients have the right to withdraw from the trial at any time for any reason. Further, the investigator has the right to withdraw a patient from the trial at any time if a withdrawal is considered in the best interest of the patient.

Patients who have ceased intervention prior to its determination will be asked to follow the scheduled controls, and data will be collected according to the protocol. Patients who choose to withdraw from the trial will be asked the reason why. However, it is emphasized that the patient is not obliged to state the cause. If a patient drops out and is unwilling to follow the protocol, permission will be asked to continue weekly phone calls to monitor potential side effects (for 4 weeks after last injection). Further, the patient will follow standard treatment for hip fracture and see the orthopedic surgeon at the regular postoperative visit.

### Outcome

**Fig. 2:**
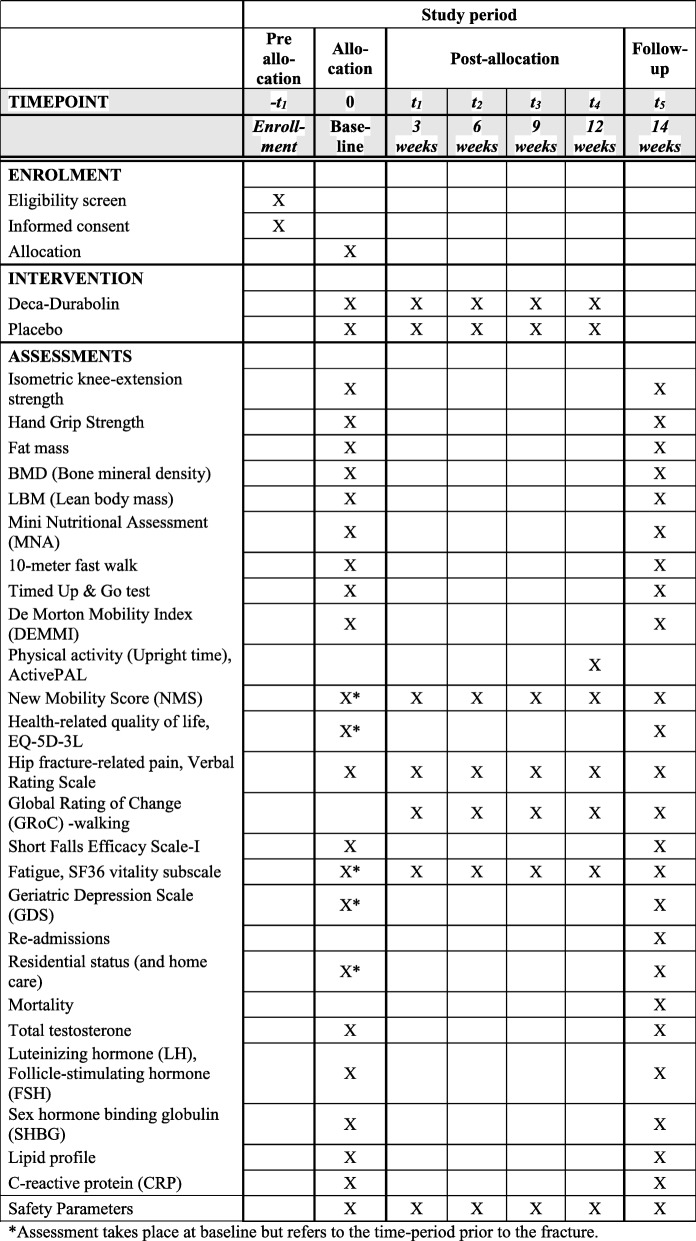
Schedule for enrollment, intervention, and outcome assessments (SPIRIT)

Outcomes will be assessed blinded at baseline and at 14 weeks after entering the trial. Safety parameters will be assessed at baseline and 3, 6, 9, 12, and 14 weeks after inclusion. Outcome assessment is carried out primarily by the project coordinator, who is an educated physiotherapist with 13 years of practical experience in orthopedics. In the project coordinator’s absence, an experienced physiotherapist trained in the protocol will conduct the assessments. In the text below, outcome parameters and time of assessment are specified, which are illustrated in Fig. 2.

The baseline assessment might extend over 2 days in order to avoid patient exhaustion, and it will be conducted during the time period from postoperative day 3 until postoperative day 10. The follow-up assessment is conducted during week 14 from time of randomization (± 7 days from time of randomization). The control every 3 weeks is conducted within 3, 6, 9, and 12 weeks (± 7 days from time of randomization).

#### Primary outcome

- Change in maximal isometric knee-extension strength (N∙m/kg) in the fractured limb (maximal voluntary torque per kilogram body mass) from baseline to the 14-week follow-up. Knee-extensor strength is chosen as the primary outcome because it is closely related to the exposure (strength training), which is what we want in this pilot trial. Hence, we consider the outcome a surrogate outcome measure for a more clinically meaningful one, such as mobility. Pertaining to this, knee-extensor strength is associated with impaired mobility [4, 33].
  Knee-extensor strength is measured using a belt-fixed handheld dynamometer (Commander Muscle Tester; JTECH Medical, Midvale, UT, USA) [3, 27, 28]. The test is conducted as described by Kronborg et al. [3] with the patient seated on the bedside, with hips and knee joint angle in 90-degree flexion and hands placed on the mattress for support. The lever arm length is measured by tape measure between the lateral epicondyle of the femur and the center of the dynamometer transducer pad placed 4 cm above the lateral malleolus of the tibial bone. Four trials must be completed, and the highest obtained value in Newtons (N) will be used for analysis. Tests are performed with standardized verbal encouragement. The isometric knee-extension strength is expressed in N∙m/kg, which is derived from the units of force measured in Newtons (N) multiplied by the corresponding lever arm measured in meters (m), divided by the weight of the patient in kilograms (kg).

#### Secondary outcomes

The following outcomes will be compared between the two groups. Unless stated otherwise, the change in values are measured from baseline until 14 weeks. Figure 2 illustrates time points for assessment of each outcome.

- Maximal isometric knee-extension strength (N∙m/kg) in the fractured limb as a percentage of the nonfractured limb. Description of the measurement method is provided under the “Primary outcome” heading.
- Maximal isometric knee-extension strength (N∙m/kg) in the nonfractured limb. Description of the measurement method is provided under the “Primary outcome” heading.
- Hand-grip strength in the dominant hand measured using a digital handheld dynamometer (Saehan Grip, DHD-1; Saehan, Changwon, Korea). Hand-grip strength will be expressed in kilograms. A standardized test protocol will be used similar to the one described by Bodilsen et al. [34].
- Fat mass (total body weight) assessed by dual x-ray absorptiometry (DEXA), expressed in kilograms. DEXA is performed as a whole-body scan and is conducted in accordance with the department’s standard procedures.
- Bone mineral density (BMD) assessed by DEXA. Registration of total body, total hip, femoral neck, and lumbar spine BMD. Expressed in g/cm^2^. Further T-score is registered. The scan is performed as a whole-body scan and is conducted in accordance with the department’s standard procedures.
- Lean body mass assessed by DEXA and expressed in kilograms. Registration of total body, legs bilaterally, and arms bilaterally. The scan is performed as a whole-body scan and is conducted in accordance with the department’s standard procedures.
- Nutritional screening using the Mini Nutritional Assessment–Short Form (MNA-SF). Total score from 0 to 14 points, high scores indicating better nutritional status. The score is frequently used for assessing nutritional status in patients with hip fracture and predicts mortality and readmissions [35, 36].
- Gait speed is assessed using the 10-m fast speed walking test, standing start. A standardized test protocol is used, and the best result of three trials is reported in meters walked per second (m/s) [28].
- The Timed Up & Go Test is performed using a four-wheeled rollator and measured in seconds. The patient has to rise from a chair, walk 3 m, turn around, walk back, and sit down [37]. A standardized instruction will be used [38].
- The de Morton Mobility Index is used for measuring mobility and consists of 15 mobility items ranging from mobility in bed to dynamic balance. The test result is a total score from 0 to 100, with 100 representing the highest level of mobility [39–41].
- Activity: Sedentary time (lying/sitting), upright time (standing/walking), steps, and transfers is measured using a body-worn accelerometer-based activity monitor (activPAL) [42]. The monitor will be attached to the thigh. The patient will wear the monitor for 1 week from the time point of the 12-week control.
- Functional level is assessed by the modified New Mobility Score [43–45]. The patients are interviewed about walking ability indoors, outdoors, and when shopping. At baseline, the score refers to the week prior to hospital admission. The total score ranges from 0 to 9. A higher score indicates greater independence.
- EQ-5D-3L is used for assessing health-related quality of life [46–48] and is administered via interview. At baseline assessment, the score refers to the time prior to the fracture.
- Hip fracture-related pain at rest and during outcome assessment is evaluated using the Verbal Rating Scale [49]. The patient is asked to rate the intensity of pain in relation to five adjectives: “no pain,” “slight pain,” “moderate pain,” “severe pain,” and “unbearable pain.” The answer is converted to a number between 0 and 4 on an ordinal scale.
- A global rating of change scale will be used for assessment of patient-perceived change in walking ability during the trial period. Patients will be asked one question related to change in mobility and have five response options ranging from much better to much worse.
- The Short Falls Efficacy Scale–International is used to measure the patient’s fear of falling (score range from 7 to 28, with higher scores indicating a greater fear of falling) [50, 51]. It is administered as an interview.
- Fatigue is assessed using the SF-36 (36-item Short Form Health Survey) vitality subscale, consisting of four items related to fatigue/energy [52, 53]. Scores range from 0 to 100 points; high score defines a more favorable health state. Administered as an interview. The baseline assessment refers to the time prior to the fracture.
- Depression is assessed using the Geriatric Depression Scale, which is administered as an interview [54, 55]. Score range, 0–15. Baseline assessment refers to the time prior to the fracture.
- Readmissions within 14 weeks will be assessed through the medical journal.
- Residential status, including home care, will be recorded by interview or medical journal.
- Mortality will be assessed through the medical journal/Danish civil register.

#### Blood tests

All blood tests are conducted in accordance with the department’s standard procedures.

- Total testosterone (nmol/L), luteinizing hormone (IU/L), follicle-stimulating hormone, (IU/L), and sex hormone-binding globulin (nmol/L).
- Lipid profile (total cholesterol, high-density lipoprotein cholesterol, low-density lipoprotein cholesterol, triglyceride) (mmol/L).
- C-reactive protein (mg/L).

#### Safety parameters

The following values are assessed: hemoglobin, hematocrit, creatinine, carbamide, sodium (Na^+^), potassium (K^+^), calcium, international normalized ratio (INR), liver tests, PSA, and glucose.

For the following parameters, safety thresholds are defined: hematocrit (safety threshold, values > 0.50), liver tests (albumin, alanine aminotransferase, γ-glutamyltransferase, bilirubin) (safety threshold, liver test values > 3 times the upper limit of normal), and PSA (safety threshold, increase to > 50%).

Other safety parameters are blood pressure (assessed using a digital blood pressure monitor, measured in mmHg) and facial hirsutism (assessed using the two face-related items of the modified Ferriman-Galwey hirsutism score, 0–8 points) [56], hoarseness (assessed through weekly interviews and hospital controls every 3 weeks), edema (assessed through weekly interviews and hospital controls every 3 weeks), and falls (a question regarding falls will be part of an interview guide used for the weekly telephone calls). Other AEs/adverse reactions (ARs) will be assessed through weekly interviews and hospital controls every 3 weeks.

#### Feasibility outcomes

Feasibility will be assed according to the following aspects: number of eligible patients, inclusion rate per month, feasibility and suitability of outcome measures, acceptability of the treatments to the patients, adherence to the treatment, retention to the scheduled controls and follow-up, and number and severity of AEs.

### Sample size

The sample size is determined on the basis of the primary outcome (change in knee-extension strength of the fractured limb) and calculated to detect a between-group difference in the change score of 0.2 N∙m/kg in favor of the intervention group using Lehr’s formula with an SD of 0.22 N∙m/kg. The difference in change scores of 0.2 N∙m/kg is defined by the authors, and it is a larger difference than what could be considered the minimal clinically important difference. Because we only wish to explore the potential of effect in this pilot trial [57], and not establish effect, we argue that it is acceptable. The SD of 0.22 N∙m/kg is obtained from a previous study [3]. Hence, we acknowledge that if this trial shows feasibility and preliminary effect of the intervention, confirmatory effect will need to be demonstrated in at least one phase III-like confirmatory trial. On the basis of this estimate, 20 patients are needed in each group using a standard of 80% power and type I error rate of 5%. Forty-eight patients are therefore planned for inclusion in the present trial to allow for an expected dropout rate of 20%. In case of dropout, new patients will be enrolled in the trial to ensure a minimum of 20 patients in each group who have completed the intervention.

### Randomization and allocation

The patients will be randomly assigned to one of the two groups by a 1:1 allocation ratio. Block randomization (blocks of 2 and 4) will be used, and patients will be stratified for type of fracture (cervical femoral versus trochanteric hip fracture) and sex. The allocation sequence is computer-generated (random number generator) by a qualified person not involved in the trial. The allocation sequence is retained in a locked cabinet by the person generating the sequence. To ensure allocation sequence concealment, sequentially numbered, opaque, sealed envelopes are used. When a person is included in the trial, the coded envelope is broken by the nurse injecting the trial medication. The envelopes contain information on allocation and a registration form used for medicine accounting. The envelopes will be retained by the nurse injecting the medication and kept in the nurse’s office, which is geographically separated from the hip fracture unit and the Department of Physiotherapy.

Information about allocation will not be revealed before all data analysis has been performed.

### Blinding procedure

The patients, healthcare providers, intervention deliverers, data collectors, and outcome assessors are all blinded to whether the patient has received trial medication or placebo. The only person not blinded is the nurse drawing the envelope and injecting the medication/placebo, but she has no other involvement in the trial. The nurse is instructed not to reveal to the patients to which intervention they are allocated. No effort will be made to blind the research hypotheses from the participants.

Blinding for the individual patient will be broken only in cases where the continued treatment of the patient requires knowledge of allocation. Twenty-four-hour access to unblinding is assured. If the code is broken, date and reason will be registered, and the envelope will be signed by the investigator.

### Data collection and management

For each patient included in the trial, an electronic case report form (CRF) will be completed in a browser-based database, Research Electronic Data Capture (REDCap). Data entered via REDCap will be stored via an encrypted connection and will meet the applicable requirements for data security. The REDCap option of validating the entered data will be used to promote the quality of data. Correction of data will be visible and accessible through REDCap’s audit trail. The audit trail will be saved equivalent to trial data. The trial data will be saved for at least 5 years as required by the Danish Data Protection Agency (journal no. AHH-2017-090, I-Suite no. 05980). The principal investigator and sponsor are responsible for managing and archiving data in accordance with the relevant legislation, including the Act on Processing of Personal Data and Health Act.

### Data monitoring

The trial will be conducted in accordance with the International Council for Harmonisation of Technical Requirements for Pharmaceuticals for Human Use (ICH) principles of good clinical practice (GCP). The project is registered with EudraCT (identifier 2017-001543-13) and is monitored by the independent GCP unit at Copenhagen University Hospital Bispebjerg. The GCP unit will monitor the project throughout the trial and assure that the trial is executed, registered, and reported according to the protocol, written standard operating procedures, GCP, and Danish legislation. Scheduled monitoring visits will be conducted throughout the trial. The first initiating visit is prior to commencement of the trial. The focus of the following visit is on monitoring the trial master file, protocol compliance, data quality, and informed consent. Further, selected trial data are monitored (e.g., inclusion, dropout, completion, primary outcome, trial medication, randomization, allocation, drug compliance, medicine accounting, AEs). No additional auditing is planned.

AEs and ARs related to the trial will be recorded in the CRF throughout the trial period, starting from the day of the first injection and ending at week 16. The relationship (causality) between the AE and the trial medication and severity will be assessed by the principal investigator or any one of the medical doctors trained in the protocol. The summary of product characteristics for Deca-Durabolin is used as a reference when assessing if a serious AR is unexpected or expected.

It should be noted that the following conditions are considered to occur often after hip fracture surgery and can lead to prolonged hospitalization: nausea, vomiting, dizziness, postoperative urine retention due to catheterization, pain or irritation in relation to the bladder due to catheterization, diarrhea, pneumonia and cardiopulmonary influence, hemoglobin (Hb) < 9.7 g/dl and consequently blood transfusion, and divergent blood tests due to surgery. These will not be registered as AEs during hospital admission. Expected pain from the operation site will not be registered as an AE during the trial period. Further, edema of the fractured limb is common in the postoperative period, and especially for patients with trochanteric fractures compared with those with cervical femoral fractures [4], and will not be reported as an AE. Laboratory test results beyond the reference interval will be recorded as an AE only if they cause a clinical action.

All AEs/ARs will be followed until stabilization by either the relevant hospital department or the patient’s general practitioner.

### Statistical analyses

Descriptive statistics will be used for presenting baseline characteristics. Continuous data will be examined for normality of distribution using Q-Q plots. Data will be presented as mean (SD) when normally distributed, otherwise as medians (q1–q3) or as frequencies with percentages.

The statistical analysis of the primary outcome will be two-sample *t* test or Wilcoxon rank-sum test as appropriate to determine systematical differences in change scores between the intervention and control groups. For the secondary outcomes, tests will be performed using either chi-square or Fisher’s exact test for categorical data or two-sample *t* test or Wilcoxon rank-sum test for continuous data. Analysis of safety parameters and feasibility endpoints will be descriptive. The level of significance will be set at *P* < 0.05, and confidence intervals (CIs) will be displayed at 95% CI around differences.

The analysis will follow the intention-to-treat principle and include all randomized patients. To create a full analysis set, missing data will be imputed using a multiple imputation technique. Secondary analysis will be conducted for both primary and secondary outcomes on the per-protocol data, where patients are excluded if they are not compliant with the trial. Compliance in relation to per-protocol analysis is defined as 75% intake of nutritional supplement, 75% completed training sessions, and 100% received injections. No interim analyses will be conducted.

### Ethics

The trial will be conducted in accordance with the principles of ICH-GCP and is monitored by the local GCP unit. The protocol is approved by the Capital Regions Research Ethics Committee (H-18004495) and the Danish Medicines Agency (EudraCT identifier 2017-001543-13). The trial is registered with the Danish Data Protection Agency (journal no. AHH-2017-090; I-Suite no. 05980).

All patients enrolled in the trial will have close contact with health professionals through weekly phone calls and hospital visits every 3 weeks. The increased attention, close contact, and strong focus on the individual patient’s well-being can in itself be perceived as positive and thus beneficial. Risks and ARs for study participants are considered to be minimal. Strength training has been widely reported as safe [3, 13, 28, 58], and the planned program ensures a familiarization phase. Occurrence of ARs in relation to trial medication are not expected, owing to the relatively short intervention period and low doses. The safety precautions in the current trial, such as close observations through weekly interviews and assessment of safety parameters every 3 weeks, are considered to be sufficient to minimize risk and discomfort to the patient. However, slight soreness at the injection site could be experienced in relation to blood sampling and injection of trial medication. DEXA scans are conducted at two time points during the trial period. Radiation exposure is minimal, approximately 0.020 mSv corresponding to 1/50th of an X-ray of the lungs, and it constitutes no health risk.

On the basis of available evidence and the safety precautions taken in the present trial, the risk to the exposed patients seems to be absolutely minimal, and we are convinced that this trial is ethical to conduct. The participants do not receive remuneration for participation in the trial.

#### Protocol amendment

The following protocol amendments were approved by the ethics committee (28 September 2018) and the Danish Medicines Agency (26 October 2018):

1. The inclusion criterion *age* was changed from ≥ 65 years to ≥ 60 years. The cut ≥ 60 years is often used internationally when referring to elderly compared with nonelderly patients with hip fracture [59]. Further, patients from 60 to 64 years old would have the same potential benefit from the intervention.
2. The exclusion criterion concerning *PSA* values was changed, so assessment of PSA is moved to the 3-week control, because a falsely elevated PSA value could be seen during admission due to urine catheterization.
3. The exclusion criterion *terminal illness* was changed to *active cancer or suspected pathological fracture*.
4. The time frame for baseline testing was changed from 6–10 days to 3–10 days due to short hospitalization for some patients.
5. The intramuscular injection of trial medication was described to be administered in the gluteal muscle of the nonfractured leg. Due to difficulties with positioning all patients lying on the side, the description has been changed so that the medication can be administered either in the gluteal muscle or in the rectus femoris

A second protocol amendment concerning extension of the inclusion period until September 2020 has been approved by the ethics committee (27 September 2019) and the Danish Medicines Agency (22 July 2019). The primary investigator and the sponsor will inform the Research Ethics Committee, the Board of Health, and the Data Protection Agency if significant changes in protocol occur.

### Dissemination

Two publications are planned for the HIP-SAP1 trial in peer-reviewed scientific journals. One is the protocol manuscript and the other is the primary trial report concerning feasibility and preliminary effects of the trial. Contributors to the trial will be offered authorship in accordance with the International Committee of Medical Journal Editors guidelines. There is no intention of using professional writers. Trial results will be published regardless of findings being positive, inconclusive, or negative. Further, results will be presented at national and international congresses. Trial participants will be notified of trial results by letter.

## Discussion

Patients with hip fracture are a vulnerable group with high morbidity and mortality. They experience large strength deficits often leading to loss of function and independence. Knowledge regarding interventions enhancing outcome and reducing loss of function in this fragile group will be of immense benefit to both the individual patient in terms of better health and quality of life and the health care system in general.

The HIP-SAP1 trial is, to our knowledge, the first trial investigating the effect of a multimodal intervention consisting of physiotherapy (functional, balance, and strength training), protein-rich nutritional supplementation, and anabolic steroid in rehabilitation following hip fracture surgery. In the literature of rehabilitation following hip fracture, there is a demand for trials exploring the effect of multimodal interventions including muscle-enhancing medicine [6, 13, 21, 24, 25].

This study will contribute useful knowledge about the feasibility, safety, and preliminary effect of such multimodal intervention. If found feasible, this pilot trial will form the basis for a larger confirmatory trial that can finally determine the effect of muscle-building medicine in the rehabilitation of elderly patients with hip fracture, and it will contribute to setting new evidence-based standards for the optimal cross-continuum treatment following hip fracture. Potentially, this will bring a greater proportion of patients back to their previous level of functioning, which might lead to reductions in new falls and fractures, need of home care, and health care costs.

The design of this pilot trial being randomized and blinded, besides clarifying the question of feasibility, will give a preliminary suggestion of effect. Further, the preliminary estimates obtained for outcome parameters in this trial will be used for sample size calculation in a confirmatory trial. Continuing to undertake a larger confirmatory trial will not be based on a statistically significant difference between groups, because the trial is not sufficiently powered, but the results of the tests will be taken into consideration along with an overall assessment of all information provided by this pilot trial.

The intervention being initiated during admission and continued for 12 weeks in the municipality mimics everyday practice, and the physiotherapy intervention is very similar to the existing standard rehabilitation offered by the municipalities, which increases external validity and will ease implementation. In regard to the nutritional component of the intervention, although an important focus area during hospitalization, it is not standard care in the municipalities to receive nutritional supplements for 12 weeks. However, the municipalities are aware of the importance of nutrition in this fragile group of patients. Some municipalities provide protein supplementation for all patients in relation to the exercise session; others conduct nutritional screening as part of the rehabilitation program, and patients in need of supplementation will be seen by a dietitian. On the basis of the current study design, we cannot make recommendations for the use of nutritional supplementation, but we will obtain information on adherence to the nutritional supplement. Knowledge obtained in this trial will inform a definitive trial.

The study is limited by narrow inclusion and exclusion criteria, and recruiting eligible patients might be difficult. The inclusion and exclusion criteria are based on previous studies using anabolic androgenic steroids in elderly patients [60, 61]. Because anabolic steroids are used in a novel field and the population is older and multimorbid, a rather conservative approach has been applied. Further, the criteria are decided on to get a comparable sample without too much “noise” from other factors that could influence outcome. If the trial is feasible and safe, less restrictive criteria might be applied for the larger confirmatory trial. The generalizability of the results of this trial will be limited to a similar population, and therefore the findings will apply only to a smaller proportion of patients with hip fracture.

## Trial status

Protocol version 7 (19 December 2019). Screening for eligible patients began 5 June 2018, and the approximate date for completion of inclusion is September 2020.

## Supplementary information

**Additional file 1.** SPIRIT Checklist.

**Additional file 2.** Strength-training exercise logs used in the municipality.

## Data Availability

MTK owns data, and all authors will have full access to the dataset. A fully patient-anonymized dataset will be made available for the scientific journal reviewing the manuscript.

## Abbreviations

AE: Adverse event
AR: Adverse reaction
CRF: Case report form
DEXA: Dual x-ray absorptiometry
GCP: Good clinical practice
MNA-SF: Mini Nutritional Assessment–Short Form
NMS: New Mobility Score
PSA: Prostate-specific antigen
PT: Physiotherapy
REDCap: Research Electronic Data Capture
Reps: Repetitions
RM: Repetition maximum
VRS: Verbal Rating Scale
