# Supplementary material for: Preliminary effect and feasibility of physiotherapy with strength training and protein-rich nutritional supplement in combination with anabolic steroids in cross-continuum rehabilitation of patients with hip fracture: protocol for a blinded randomized controlled pilot trial (HIP-SAP1 trial)
Source: Trials. 2019 Dec 23;20:763. doi: 10.1186/s13063-019-3845-y (PMC6929304; doi:10.1186/s13063-019-3845-y)
Supplement: Supplementary file 2 — Additional file 2. Strength-training exercise logs used in the municipality. [file 13063_2019_3845_MOESM2_ESM.pdf]

Training schedule 1: Seated leg press (Week 1 – 6)

Name____ Date start of training____________________

Both legs are trained bilaterally

Date	Planned training	Pain at rest 0-4	Sets	Repetitions performed	Kilograms "lifted"	Pain during training 0-4	No/limited training
/	3 x 15 reps with 15RM		1.				
			2.				
			3.				
/	3 x 15 reps with 15RM		1.				
			2.				
			3.				
/	3 x 15 reps with 15RM		1.				
			2.				
			3.				
/	3 x 15 reps with 15RM		1.				
			2.				
			3.				
/	3 x 12 reps with 12RM		1.				
			2.				
			3.				
/	3 x 12 reps with 12RM		1.				
			2.				
			3.				
/	3 x 12 reps with 12RM		1.				
			2.				
			3.				
/	3 x 12 reps with 12RM		1.				
			2.				
			3.				
/	3 x 10 reps with 10RM		1.				
			2.				
			3.				
/	3 x 10 reps with 10RM		1.				
			2.				
			3.				
/	3 x 10 reps with 10RM		1.				
			2.				
			3.				
/	3 x 10 reps with 10RM		1.				
			2.				
			3.				

Instructions:
Take as many repetitions (reps) as possible in each set.

If number of reps varies with more than 3 according to the planned, then adjust the load in the next set.

Both the concentric and eccentric phase is performed slowly and controlled.

Min. 1-minute rest between sets.

Verbal Rating Scale for pain: Fractured hip area:
0: No pain
1: Slight pain
2: Moderate pain
3: Severe pain
4: Unbearable pain

No/limited training:

Report reason if training / sets are not completed as planned:

1. Pain fractured hip area
2. Fatigue / exhaustion
3. Cancellation (report cause in scheme)
4. Training cancelled by center
5. Other (report cause in scheme)

Modified by Signe Hulsbæk, PT, PhD-student, 2018 after Morten Tange Kristensen, PT, Senior Researcher, Departments of Physical Therapy and Orthopedic Surgery, Hvidovre Hospital, 2008.

Training schedule: Seated leg press (Week 7 – 12)

Name____ Date start of training____________________

Both legs are trained bilaterally

Date	Planned training	Pain at rest 0-4	Sets	Repetitions performed	Kilograms "lifted"	Pain during training 0-4	No/limited training
/	3 x 10 reps with 15RM		1.				
			2.				
			3.				
/	3 x 10 reps with 15RM		1.				
			2.				
			3.				
/	3 x 10 reps with 15RM		1.				
			2.				
			3.				
/	3 x 10 reps with 15RM		1.				
			2.				
			3.				
/	3 x 10 reps with 12RM		1.				
			2.				
			3.				
/	3 x 10 reps with 12RM		1.				
			2.				
			3.				
/	3 x 10 reps with 12RM		1.				
			2.				
			3.				
/	3 x 10 reps with 12RM		1.				
			2.				
			3.				
/	3 x 10 reps with 10RM		1.				
			2.				
			3.				
/	3 x 10 reps with 10RM		1.				
			2.				
			3.				
/	3 x 10 reps with 10RM		1.				
			2.				
			3.				
/	3 x 10 reps with 10RM		1.				
			2.				
			3.				

Instructions:
Take as many repetitions (reps) as possible in each set.

If number of reps varies with more than 3 according to the planned, then adjust the load in the next set.

Both the concentric and eccentric phase is performed slowly and controlled.

Min. 1-minute rest between sets.

Verbal Rating Scale for pain: Fractured hip area:
0: No pain
1: Slight pain
2: Moderate pain
3: Severe pain
4: Unbearable pain

No/limited training:

Report reason if training / sets are not completed as planned:

1. Pain fractured hip area
2. Fatigue / exhaustion
3. Cancellation (report cause in scheme)
4. Training cancelled by center
5. Other (report cause in scheme)

Training schedule 1: Knee-extension (Week 1 – 6)

Name____ Date start of training____________________

<u>Both legs are trained, unilaterally – non-fractured leg first</u>

Date	Planned training	Pain at rest 0-4	Sets	Repetitions performed		Kilograms "lifted"		Pain during training 0-4	No/limited training
				Non-fractured	Fractured	Non-fractured	Fractured		
/	3 x 15 reps with 15RM		1.						
			2.						
			3.						
/	3 x 15 reps with 15RM		1.						
			2.						
			3.						
/	3 x 15 reps with 15RM		1.						
			2.						
			3.						
/	3 x 15 reps with 15RM		1.						
			2.						
			3.						
/	3 x 12 reps with 12RM		1.						
			2.						
			3.						
/	3 x 12 reps with 12RM		1.						
			2.						
			3.						
/	3 x 12 reps with 12RM		1.						
			2.						
			3.						
/	3 x 12 reps with 12RM		1.						
			2.						
			3.						
/	3 x 10 reps with 10RM		1.						
			2.						
			3.						
/	3 x 10 reps with 10RM		1.						
			2.						
			3.						
/	3 x 10 reps with 10RM		1.						
			2.						
			3.						
/	3 x 10 reps with 10RM		1.						
			2.						
			3.						

Instructions:
Take as many repetitions (reps) as possible in each set.

If number of reps varies with more than 3 according to the planned, then adjust the load in the next set.

Both the concentric and eccentric phase is performed slowly and controlled.

Min. 1-minute rest between sets.

Verbal Rating Scale for pain:
Fractured hip area:
0: No pain
1: Slight pain
2: Moderate pain
3: Severe pain
4: Unbearable pain

No/limited training:

Report reason if training / sets are not completed as planned:

1. Pain fractured hip area
2. Fatigue / exhaustion
3. Cancellation (report cause in scheme)
4. Training cancelled by center
5. Other (report cause in scheme)

Modified by Signe Hulsbæk, PT, PhD-student, 2018 after Morten Tange Kristensen, PT, Senior Researcher, Departments of Physical Therapy and Orthopedic Surgery, Hvidovre Hospital, 2008.

Training schedule: Knee-extension (Week 7 – 12)

Name____ Date start of training____________________

Both legs are trained, unilaterally – non-fractured leg first

Date	Planned training	Pain at rest 0-4	Sets	Repetitions performed		Kilograms "lifted"		Pain during training 0-4	No/limited training
				Non-fractured	Fractured	Non-fractured	Fractured		
/	3 x 10 repetitions with 10RM		1.						
			2.						
			3.						
/	3 x 10 repetitions with 10RM		1.						
			2.						
			3.						
/	3 x 10 repetitions with 10RM		1.						
			2.						
			3.						
/	3 x 10 repetitions with 10RM		1.						
			2.						
			3.						
/	3 x 10 repetitions with 10RM		1.						
			2.						
			3.						
/	3 x 10 repetitions with 10RM		1.						
			2.						
			3.						
/	3 x 10 repetitions with 10RM		1.						
			2.						
			3.						
/	3 x 10 repetitions with 10RM		1.						
			2.						
			3.						
/	3 x 10 repetitions with 10RM		1.						
			2.						
			3.						
/	3 x 10 repetitions with 10RM		1.						
			2.						
			3.						
/	3 x 10 repetitions with 10RM		1.						
			2.						
			3.						
/	3 x 10 repetitions with 10RM		1.						
			2.						
			3.						

Instructions:
Take as many repetitions (reps) as possible in each set.

If number of reps varies with more than 3 according to the planned, then adjust the load in the next set.

Both the concentric and eccentric phase is performed slowly and controlled.

Min. 1-minute rest between sets.

Verbal Rating Scale for pain: Fractured hip area:
0: No pain
1: Slight pain
2: Moderate pain
3: Severe pain
4: Unbearable pain

No/limited training:

Report reason if training / sets are not completed as planned:

1. Pain fractured hip area
2. Fatigue / exhaustion
3. Cancellation (report cause in scheme)
4. Training cancelled by center
5. Other (report cause in scheme)
